# Supplementary material for: Carob pod polyphenols suppress the differentiation of adipocytes through posttranscriptional regulation of C/EBPβ
Source: PLoS One. 2021 Mar 8;16(3):e0248073. doi: 10.1371/journal.pone.0248073 (PMC7939365; doi:10.1371/journal.pone.0248073)
Supplement: S1 Raw images — (PDF) [file pone.0248073.s001.pdf]

S1. Fig. 11B (C/EBPβ 48 h)

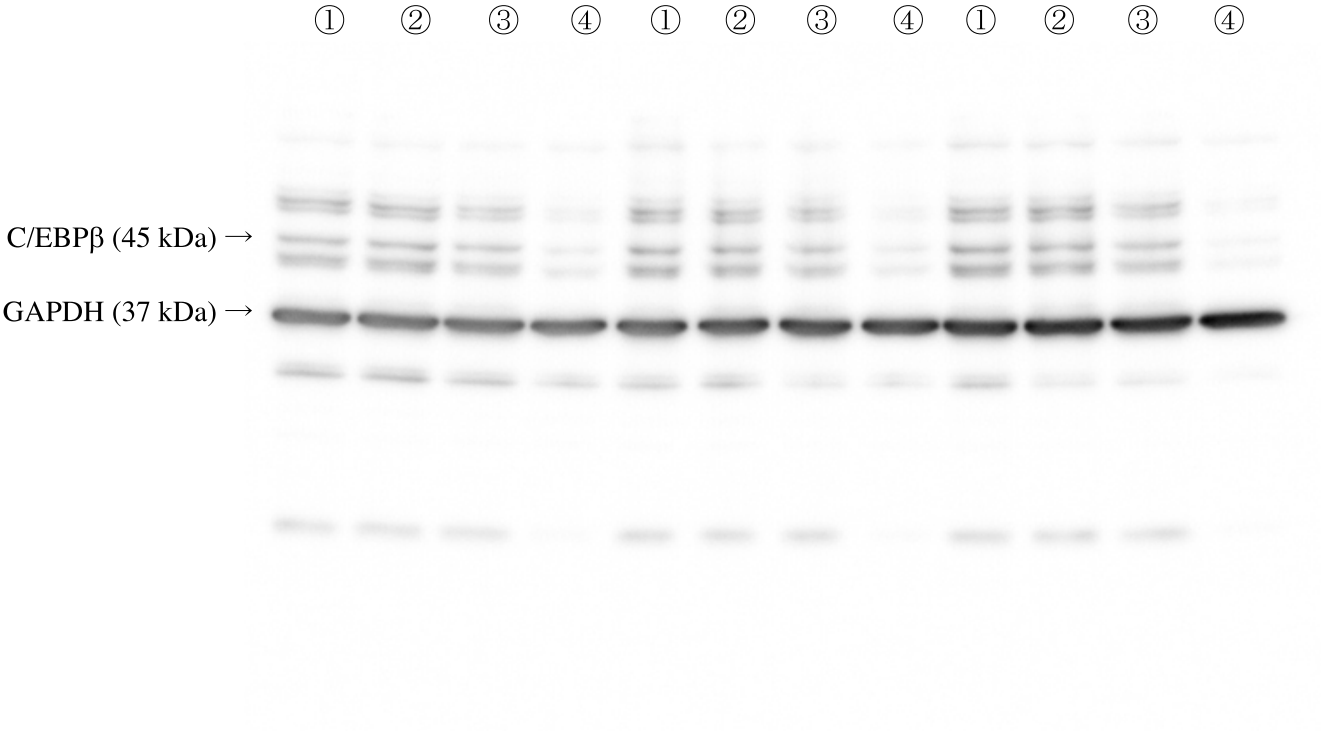

① Control, ② CCP (light) 50 μg/ml, ③ CCP (light) 100 μg/ml , ④ Undifferentiation

S1. Fig. 12C (PPARγ 48 h)

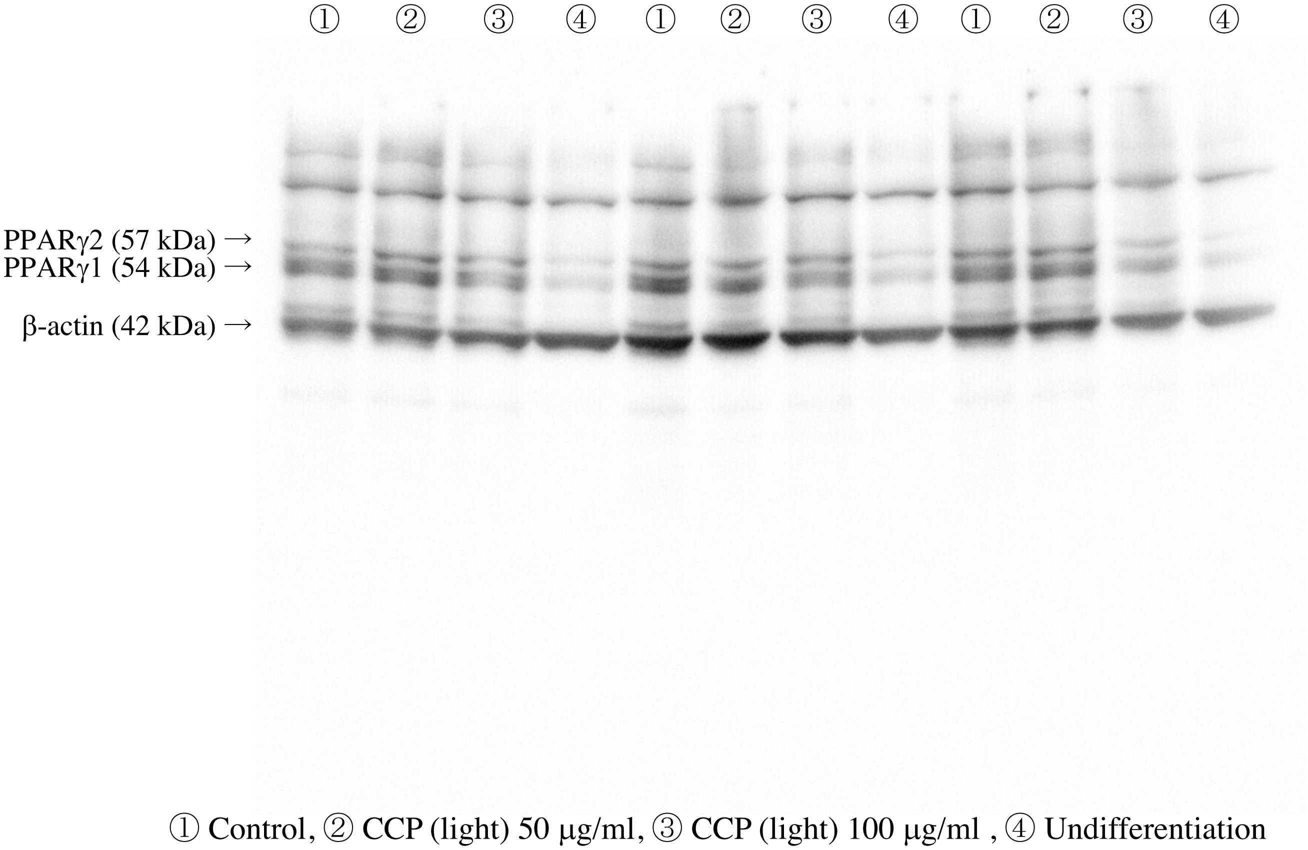

① Control, ② CCP (light) 50 μg/ml, ③ CCP (light) 100 μg/ml , ④ Undifferentiation

S1. Fig. 12D (PPARγ 72 h)

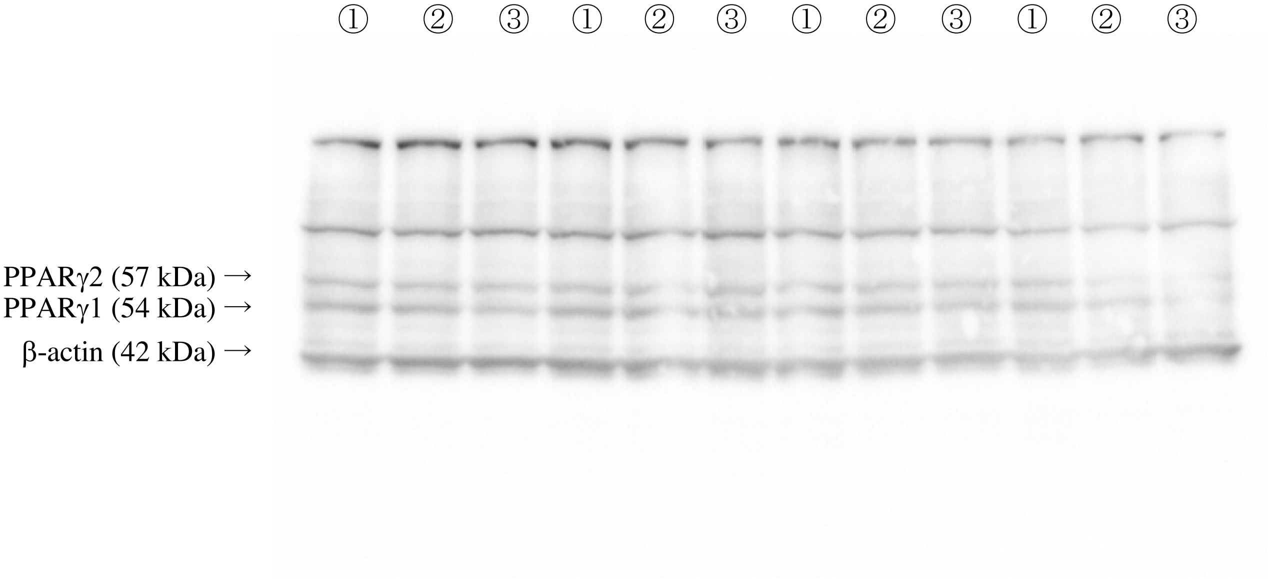

① Control, ② CCP (light) 50 μg/ml, ③ CCP (light) 100 μg/ml

S1. Fig. 13C (C/EBPα 24 h)

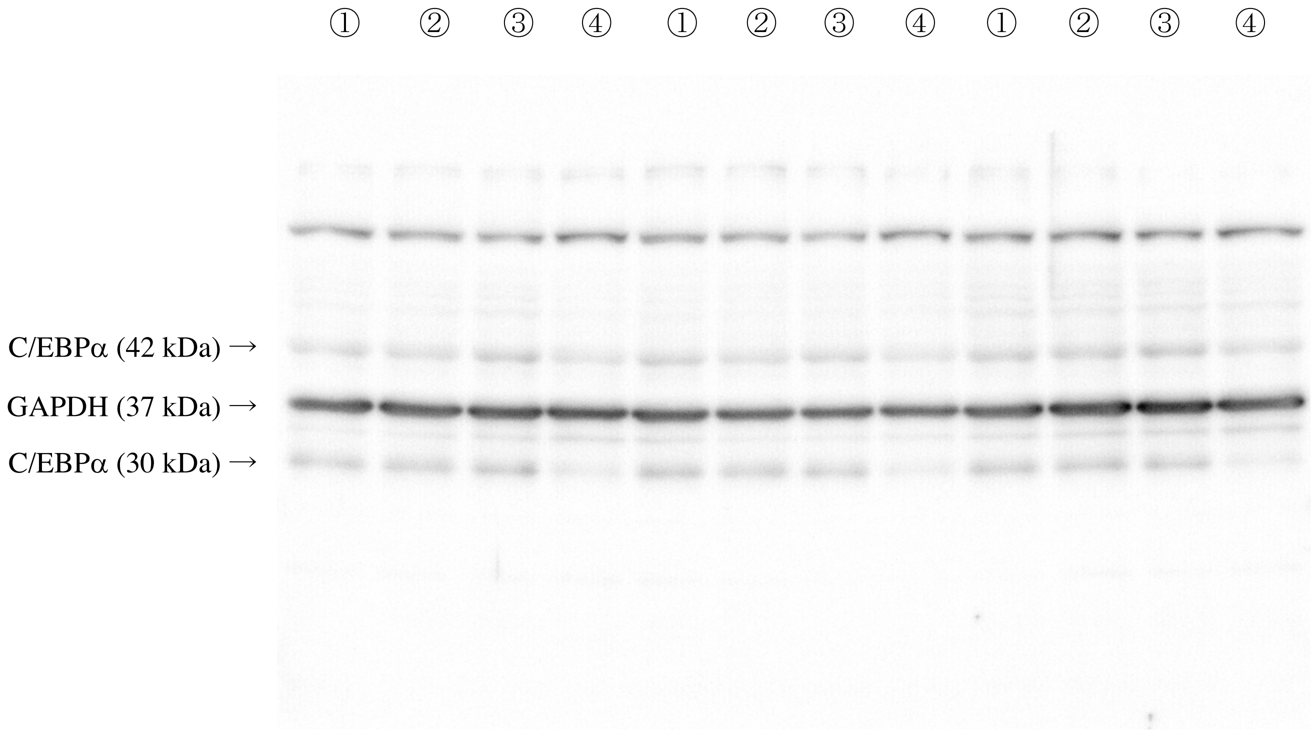

① Control, ② CCP (light) 50 μg/ml, ③ CCP (light) 100 μg/ml , ④ Undifferentiation

S1. Fig. 13D (C/EBPα 48 h)

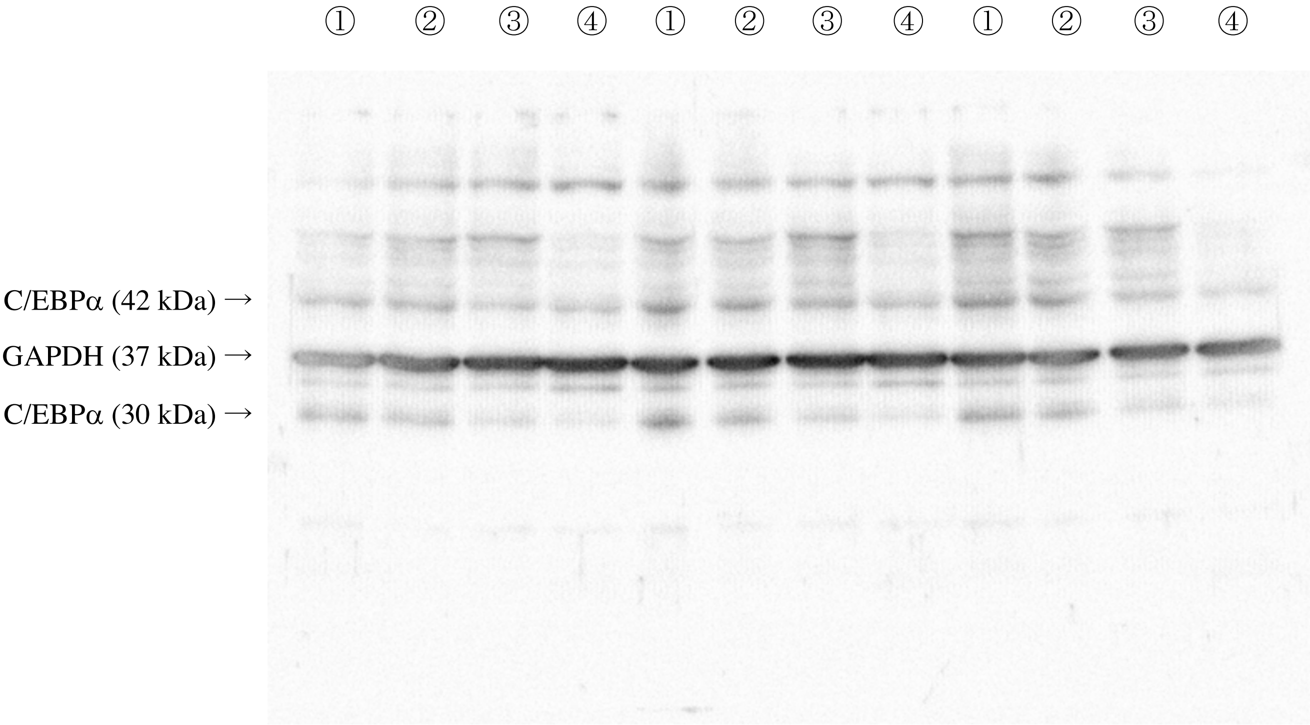

① Control, ② CCP (light) 50 μg/ml, ③ CCP (light) 100 μg/ml , ④ Undifferentiation
